# Metformin Inhibits the Progression of Pancreatic Cancer Through Regulating miR‐378a‐3p/VEGFA/RGC‐32 Axis

**DOI:** 10.1002/cam4.70446

**Published:** 2024-11-28

**Authors:** Jinli He, Yixing Luo, Ying Ding, Liang Zhu

**Affiliations:** ^1^ Department of Gastroenterology The First Affiliated Hospital, Jiangxi Medical College, Nanchang University Nanchang China; ^2^ Department of Medical Cosmetology The Second Affiliated Hospital, Jiangxi Medical College, Nanchang University Nanchang China

**Keywords:** Met, miR‐378a‐3p, pancreatic cancer, VEGFA

## Abstract

**Background:**

Pancreatic cancer (PC) is a major contributor to global cancer‐related mortality. While the inhibitory effect of metformin (Met) on PC has been reported, the underlying mechanism remains elusive.

**Methods:**

We established BxPC‐3 cell models with miR‐378a‐3p and VEGFA knockdown. The expression of miR‐378a‐3p, VEGFA, and RGC‐32 in PC and normal tissues was analyzed using GEPIA, TCGA databases. Cell proliferation, invasion, migration, and apoptosis were assessed through CCK8, Transwell, wound healing, and flow cytometry.

**Results:**

Significantly lower expression of miR‐378a‐3p was observed in PC tissues and cells. Knockdown of miR‐378a‐3p reversed the impact of Met on cell viability in PANC‐1 and BxPC3. VEGFA emerged as a potential regulator in PC and a downstream target of miR‐378a‐3p. The interaction between VEGFA and RGC‐32 played a crucial role in PC regulation. Knockdown of VEGFA substantially reversed the impact of miR‐378a‐3p inhibitor on tumor growth and the epithelial‐mesenchymal transition (EMT) process. Moreover, knockdown of VEGFA effectively countered the influence of miR‐378a‐3p inhibitor on cell viability and the EMT process in BxPC3 cells.

**Conclusions:**

Met exerted inhibitory effects on PC through the miR‐378a‐3p/VEGFA/RGC‐32 pathway. Strategies targeting the miR‐378a‐3p/VEGFA/RGC‐32 axis represent a novel avenue for the prevention and treatment of PC.

**Abbreviations**
IHC: immunohistochemical staining
Met: Metformin
miRNAs: microRNAs
PLGF: placental growth factor
VEGF: vascular endothelial growth factor

## 1 Introduction

Pancreatic cancer (PC) is a highly malignant tumor characterized by a high degree of malignancy, early propensity for metastasis, and extremely poor prognosis [1]. Early diagnosis is challenging, and most patients lose the optimal surgical window due to local tumor infiltration and/or distant metastasis when the diagnosis is confirmed. Conventional chemotherapy has limited efficacy, with a 5‐year survival rate of less than 10% [2].

Metformin (Met) is a widely used to manage type 2 diabetes by lowering blood glucose levels, and improving insulin sensitivity. Met exhibits a pleiotropic nature, including cardiovascular protection, anti‐aging effects, weight management, neuroprotective effects, and anti‐tumor. It was reported that Met activates AMP‐activated protein kinase (AMPK), and inhibit mammalian target of rapamycin (mTOR) pathway, which is crucial for tumor cell growth and proliferation [3]. In addition, Met disrupts metabolic pathways, particularly mitochondrial respiration, leading to reduced energy availability and cancer cell death [4]. In recent years, multiple studies have confirmed that Met can serve as a novel adjuvant therapy for PC, significantly prolonging patient survival [5]. However, the treatment mechanism of Met in PC is not yet elucidated. In‐depth exploration and clarification of the mechanisms underlying Met therapeutic effects in PC can provide a theoretical foundation for its improved clinical application.

MicroRNAs (miRNAs) are a class of small endogenous non‐coding RNAs that play a crucial role in cell proliferation and differentiation by inhibiting the translation or inducing the degradation of target mRNAs [6]. They also play important roles in regulation of various tumors. miRNAs can act as either oncogenes or tumor suppressors, and their dysregulation is often associated with tumorigenesis. miRNAs regulate gene expression at the post‐transcriptional level to affect tumor development, and the main mechanisms include mRNA degradation, translational repression, and miRNA sponges [7]. Previous studies have indicated that Met exerts its anti‐tumor effects by directly regulating the expression of miRNAs [8]. Researches have shown that miR‐378a‐3p is downregulated in lung cancer, ovarian cancer, and colorectal cancer, and it can inhibit the proliferation of tumor cells [9]. Similarly, in PC, miR‐378a‐3p is also downregulated, suggesting that miR‐378a‐3p may be involved in the mechanism of PC development [10]. Whether Met can influence the progression of PC by regulating miR‐378a‐3p is not yet known.

Vascular endothelial growth factor‐A (VEGFA) plays a crucial role in the physiological and pathological processes of angiogenesis. VEGFA is highly expressed in most human tumors and is closely associated with tumor invasiveness, vascular density, metastasis, recurrence, and prognosis [11]. However, the specific mechanisms by which Met regulates the expression of VEGFA require further investigation.

RGC‐32 is a complement activation gene expressed in various organ tissues, including the pancreas, participating in the regulation of cell cycle and cell differentiation [12]. Recent studies have indicated that RGC‐32 is associated with lipid metabolism and insulin resistance. RGC‐32 mediates high‐fat diet‐induced obesity and plays a significant role in the mechanism of insulin resistance [13]. Moreover, insulin can induce the expression of RGC‐32 through the NF‐κB signaling pathway [14]. Considering that Met acts by improving insulin resistance and regulating lipid metabolism, we hypothesize that RGC‐32 is likely one of the downstream targets of Met.

In this research, we established BxPC‐3 cell lines with knockdown of miR‐378a‐3p, VEGFA. Through comprehensive analysis using GEPIA, TCGA, CancerMiRNome, and dbDEMC databases, we investigated the expression of miR‐378a‐3p, VEGFA, and RGC‐32 in PC tissues and adjacent tissues, exploring their probability as tumor markers. Our findings support the hypothesis that Met may regulate the occurrence and development of PC through the miR‐378a‐3p/VEGFA/RGC‐32 pathway. This provides a novel strategy for the prevention and treatment of PC.

## 2 Materials and Methods

### 2.1 Cell Culture

Human pancreatic ductal epithelial (HPDE) cells and PC cell lines, including BxPC‐3, PANC‐1, SW1990, and MIA PaCa‐2, were obtained from the American Type Culture Collection (ATCC, USA). The cells were incubated in Dulbecco's modified Eagle's medium (DMEM, #12491015, Gibco, USA), supplemented with 5% fetal bovine serum (FBS, #26010066, Gibco, USA), 50 μg/mL streptomycin (#ST487, Beyotime, China), and 50 IU/mL penicillin (#V900929, Sigma, USA). The cell culture was kept in a humidified incubator at 37°C with 5% CO_2_.

### 2.2 Cell Transfection

miR‐378a‐3p inhibitors, sh‐VEGFA, and corresponding control vectors were designed and purchased from Shanghai Biosciences, Shanghai, China. These components were diluted and mixed gently in Opti‐MEM (#31985062, Gibco, USA). Lipofectamine 2000 (#11668019, Invitrogen, USA) was also diluted at a 1:50 ratio and mixed gently. The dilutions were incubated at room temperature for 20 min. The existing culture medium in the wells was aspirated, the wells were washed twice with phosphate buffer saline (PBS), and replaced with Opti‐MEM reduced serum medium before adding the transfection mixture. Following gentle shaking, the plates were incubated for further cultivation. After 6 h, the medium was replaced with fresh cell culture medium.

### 2.3 Establishment of a Mouse PC Model

Nude mice at the age of 4–6 weeks were subcutaneously injected with BxPC‐3 cells (2 × 10^7^ cells per mouse) in the dorsal region. Approximately 1 week later, palpable subcutaneous tumor masses were observed. Once the tumors reached measurable sizes, the mice were grouped for drug treatment. The animals in the control group were treated with physiological saline, while the remaining three groups of nude mice were treated with Met (200 mg/kg) by intraperitoneal injection once a day, sustaining over a period of 4 weeks. Two weeks after inoculation, the mice in the group Met+inhibitor received intravenous injections of miR‐378a‐3p inhibitor (5 × 10^9^ pfu), and the mice in the group Met+inhibitor+sh‐VEGF (5 × 10^9^ pfu) received sh‐VEGF and miR‐378a‐3p inhibitor. The other two groups received injections of the same dose of negative control adenovirus recombinant vectors. Tumor sizes were detected every 5 days. After 4 weeks, the mice were euthanized, and tumor weights were measured for subsequent studies. All procedures and experimental designs were conducted in accordance with the guidelines of the Animal Ethics Committee of the First Affiliated Hospital, Jiangxi Medical College, Nanchang University.

### 2.4 Immunohistochemical Staining (IHC)

Tissues were fixed in 4% paraformaldehyde (#30525‐89‐4, Sigma, USA) overnight, dehydrated, and embedded in paraffin. The embedded tissues were sectioned continuously at 8 μm thickness. The sections were placed in a 60°C incubator for 6 h. Deparaffinization was performed using xylene, and then rehydration was carried out. The sections were rinsed with running tap water for 5 min, followed by inactivation of endogenous peroxidases using endogenous peroxidase inhibitor. Blocking was achieved using goat serum for 30 min. The primary antibody was incubated overnight. After three 5‐min washes with PBS, the secondary antibody was incubated for 30 min, followed by another three 5‐min PBS washes. DAB reagent was applied for color development, and hematoxylin was used for nuclear counterstaining. The sections were then rinsed with tap water for 5 min, blued with saturated lithium carbonate, rinsed again with tap water, and dehydrated in a graded ethanol series (75%, 95%, 100%) for 3 min each. Cover slips were applied for observation.

### 2.5 Cell Cycle Analysis

Cells were plated (5 × 10^5^ cells each well) in a 6‐well culture plate and treated according to the intervention measures mentioned above. After 48 h, the cells were harvested using trypsin digestion. The cells were washed with cold PBS. Subsequently, the cells were fixed in cold 70% ethanol and stored at 4°C for 12 h. Following a final centrifugation, the cells were incubated with 10 μL of RNase A (10 mg/mL) for 30 min. After inactivation of RNase A on ice, the cells were stained with 10 μL of propidium iodide (PI, 1 mg/mL). The stained cells were then incubated for 30 min and subjected to flow cytometry for cell cycle analysis.

### 2.6 Cell Apoptosis Measurement

Cells were harvested using trypsin digestion, centrifuged at 1000 rpm for 5 min. The cell pellet was mixed with pre‐chilled PBS and centrifuged again. The cells were then resuspended in binding buffer (100 μL), and FITC‐labeled Annexin‐V (10 μL, 20 μg/mL, Beyotime, #C1062L, China) was added. The mixture was kept in the dark (30 min). Following the addition of 5 μL of PI, the cells were kept in the dark for 5 min. Then, binding buffer (400 μL) was added, and flow cytometry was performed within 1 h.

### 2.7 Western Blotting

Proteins in the cells and tissues were extracted with RIPA lysis buffer (#R0278, Sigma, US). Protein concentration was measured with BCA kit (#A045‐4‐1, Nanjing Jiancheng Bioengineering Institute, Nanjing, China), and same amounts of protein (15 μg) were resolved on 10% SDS–PAGE gels. Subsequently, proteins were transferred to a PVDF membrane. After blocking with 5% skim milk in TBST (Beyotime, #P0222, China) for 3 h, the membranes were incubated with primary antibodies at 4°C, followed by a 3‐h incubation with secondary antibodies. Target genes were detected using an enhanced chemiluminescence kit from Thermo Fisher, and band analysis was conducted using ImageJ software. The antibodies used were purchased from Abcam, and are listed below. VEGFA (#ab1316), GAPDH (#ab9485), RGC‐32 (#ab221098), N‐cadherin (#ab245117), Vimentin (#ab8978), E‐cadherin (#ab231303), p‐PI3K (#ab138364), PI3K (#ab302958), p‐AKT (#ab38449), AKT (#ab179463), β‐actin (#ab8226).

### 2.8 CCK8 Assay

Cells were plated (1 × 10^4^ cells/well) in a 96‐well plate. The final concentration of Met solution was 20 mM. After 48 h, fresh medium (100 μL) was added to each well. Then, CCK‐8 reagent (10 μL, Beyotime, #C0038, China) was added, and the cells were incubated for 4 h before detecting absorbance at 450 nm.

### 2.9 Transwell Assay

BxPC‐3 and PANC‐1 cells were plated (1 × 10^5^ cells/well) in the upper chamber of a 24‐well Transwell plate (containing Matrigel for invasion assays, #3422, BD Bioscience, USA). Serum‐free medium was added to the upper chamber, while the lower chamber was added with medium containing FBS. After 48 h, the chambers were washed with PBS twice, fixed with 4% paraformaldehyde for 30 min, and washed with PBS for three times. Non‐invasive cells were removed from the upper membrane surface using a cotton swab, and invasive cells were stained with 0.1% crystal violet for 15 min. After washing with PBS, the membranes were air‐dried, and cells were observed under a microscope at 200× magnification. Images were captured from five randomly selected fields, and the average cell count per field was used for statistical analysis.

### 2.10 Wound Healing Assay

The cells (1 × 10^5^ cells/well) were plated in a 6‐well culture plate. Upon reaching 90% confluence, a sterile 200 μL pipette tip was applied to make a scratch in the cell monolayer. The cells were washed with PBS twice to remove detached cells, and images were captured under a microscope to document the wound area. The scratch area was quantified at 0 and 24 h using ImageJ.

### 2.11 Bioinformatic Analysis

Gene expression, miRNA expression, survival analyses were performed with GEPIA (http://gepia.cancer‐pku.cn/), TCGA (https://www.cancer.gov/about‐nci/organization/ccg/research/structural‐genomics/tcga), CancerMiRNome (http://bioinfo.jialab‐ucr.org/CancerMIRNome/), and dbDEMC (https://www.biosino.org/dbDEMC/index) database.

### 2.12 Clinical Samples Collection

A total of 116 specimens diagnosed with PC from December 2019 to October 2023 were obtained from the clinical biospecimen repository. Tumor tissues were excised during surgery and preserved in liquid nitrogen. Clinical and pathological information of the patients was retrieved from the hospital's case system. The study followed the ethical standards of the Helsinki Declaration, with approval from the ethics committee of the First Affiliated Hospital, Jiangxi Medical College, Nanchang University.

### 2.13 Co‐Immunoprecipitation

Cell lysis was performed by adding 600 μL of pre‐cooled lysis buffer to scrape the cells into a 1.5 mL EP tube. Subsequently, 100 μL of magnetic beads were added to the EP tube. After magnetic bead adsorption, the supernatant was removed. Next, 1 mL of PBST was added to wash the magnetic beads, followed by thorough resuspension and magnetic bead adsorption to remove the supernatant. Then, 200 μL of diluted IP antibody (10 μg of IP antibody) was added to the magnetic beads and thoroughly resuspended. The mixture was incubated on a rolling shaker at room temperature for 30 min. After magnetic bead adsorption, the supernatant was removed. Cell protein lysis buffer (500 μL) was added, and the solution was homogenized using a pipette gun. The mixture was incubated on a rolling shaker at room temperature for 1 h, followed by incubation at 4°C overnight. After magnetic bead adsorption, the supernatant was removed. The magnetic beads were washed three times with PBST. After a quick centrifugation for 30 s, all beads on the wall were collected, and the remaining liquid was aspirated using a magnetic stand. Subsequently, 40 μL of 1× SDS‐PAGE loading buffer was added, mixed, and incubated at 70°C in a metal bath for 10 min. The SDS‐PAGE loading buffer was then transferred to a new EP tube for detection samples, and the samples were loaded directly for electrophoresis.

### 2.14 Statistical Analysis

The data are shown as mean ± standard deviation. Analysis was conducted using SPSS software version 22. Unpaired Student's *t* test and the Wilcoxon test were performed to compare the normal or non‐normal distribution of data. *p* < 0.05 was believed to be statistically significant.

## 3 Results

### 3.1 Significant Lower Expression of miR‐378a‐3p Was Observed in the PC Tissues and Cells

**FIGURE 1:**
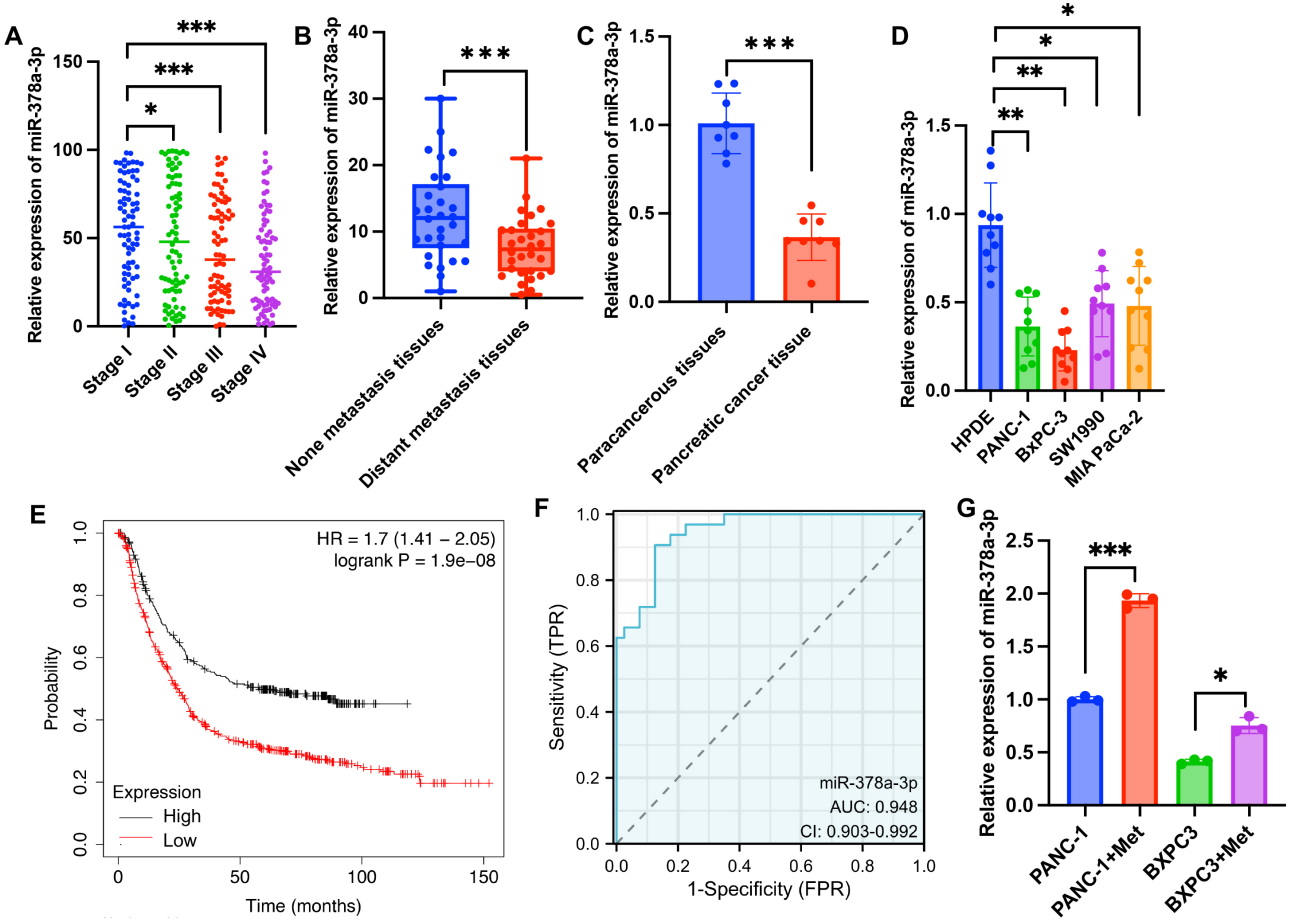
Significant lower expression of miR‐378a‐3p was observed in the PC tissues and cells. (A) The relative expression levels of miR‐378a‐3p in different stages of PC tissues based on TCGA database. (B, C) The relative expression levels of miR‐378a‐3p in distant metastasis, none distant metastasis, PC, or paracancerous tissues. (D) The relative expression levels of miR‐378a‐3p in PC cell lines and normal cell line. (E) Kaplan–Meier survival analysis based on miR‐378a‐3p expression PC patients from the TCGA database. (F) Receiver operating characteristic curve analysis is presented for miR‐378a‐3p. (G) The mRNA levels of miR‐378a‐3p were presented after Met treatment. Significance levels are denoted as (***, *p* < 0.001; **, *p* < 0.01; *, *p* < 0.05).

Using the GEPIA, TCGA, CancerMiRNome, and dbDEMC databases, we found that miR‐378a‐3p demonstrated a negative correlation with the malignancy and staging of PC, with significantly lower expression in tissues associated with distant metastasis (Figure 1A,B). Additionally, compared to adjacent cancer tissues and normal pancreatic cells, miR‐378a‐3p exhibits a marked downregulation in both PC tissues and PC cells (Figure 1C,D). The diminished expression of miR‐378a‐3p is indicative of a poorer prognosis and survival rate (Figure 1E). MiR‐378a‐3p emerges as a promising prognostic marker for PC, with an AUC reaching 0.948 (Figure 1F). Furthermore, we observed that Met significantly enhanced the expression of miR‐378a‐3p in PANC‐1 and BxPC3 cells (Figure 1G).

### 3.2 Knockdown of miR‐378a‐3p Reversed the Influence of Met on Cell Viability of PANC‐1 and BxPC3

**FIGURE 2:**
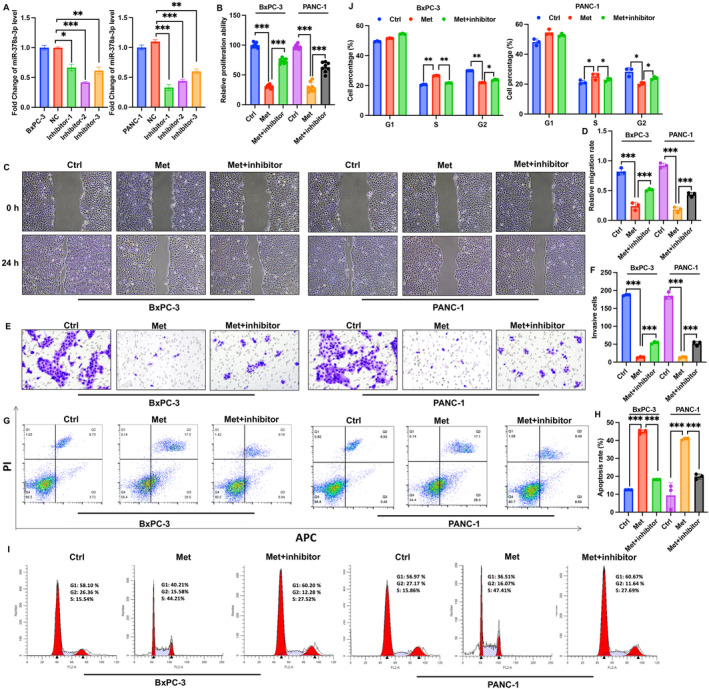
Knockdown of miR‐378a‐3p reversed the influence of Met on cell viability of PANC‐1 and BxPC3. (A) Established a miR‐378a‐3p knockdown models using PANC‐1 and BxPC3 cell lines. (B) Employed the CCK8 method to assess cell proliferation. (C, D) Investigated cell migration through a scratch assay. (E, F) Examined cell invasion using the Transwell method. (G, J) Evaluated cell apoptosis and cell cycle through flow cytometry. Significance levels are denoted as (***, *p* < 0.001; **, *p* < 0.01; *, *p* < 0.05).

To further validate the role of miR‐378a‐3p in the development of PC, we established a miR‐378a‐3p knockdown model in PANC‐1 and BxPC3 cell lines (Figure 2A). We observed that Met significantly inhibited the proliferation, migration, and invasion of PANC‐1 and BxPC3 cells while promoting cell apoptosis (Figure 2B–H). However, following transfection with the miR‐378a‐3p inhibitor, cell proliferation, migration, and invasion capabilities were significantly enhanced, while apoptosis rates decreased compared with group Met. Additionally, Met markedly increased the proportion of cells in the S phase and decreased the proportion in the G2 phase, indicating that Met could arrest cells in the S phase, thereby reducing cell proliferation (Figure 2I,J). Nevertheless, the miR‐378a‐3p inhibitor reversed the effects of Met on PANC‐1 and BxPC3 cells (Figure 2I,J).

### 3.3 VEGFA Is the Potential Regulator Molecule of PC and the Downstream Target of miR‐378a‐3p

**FIGURE 3:**
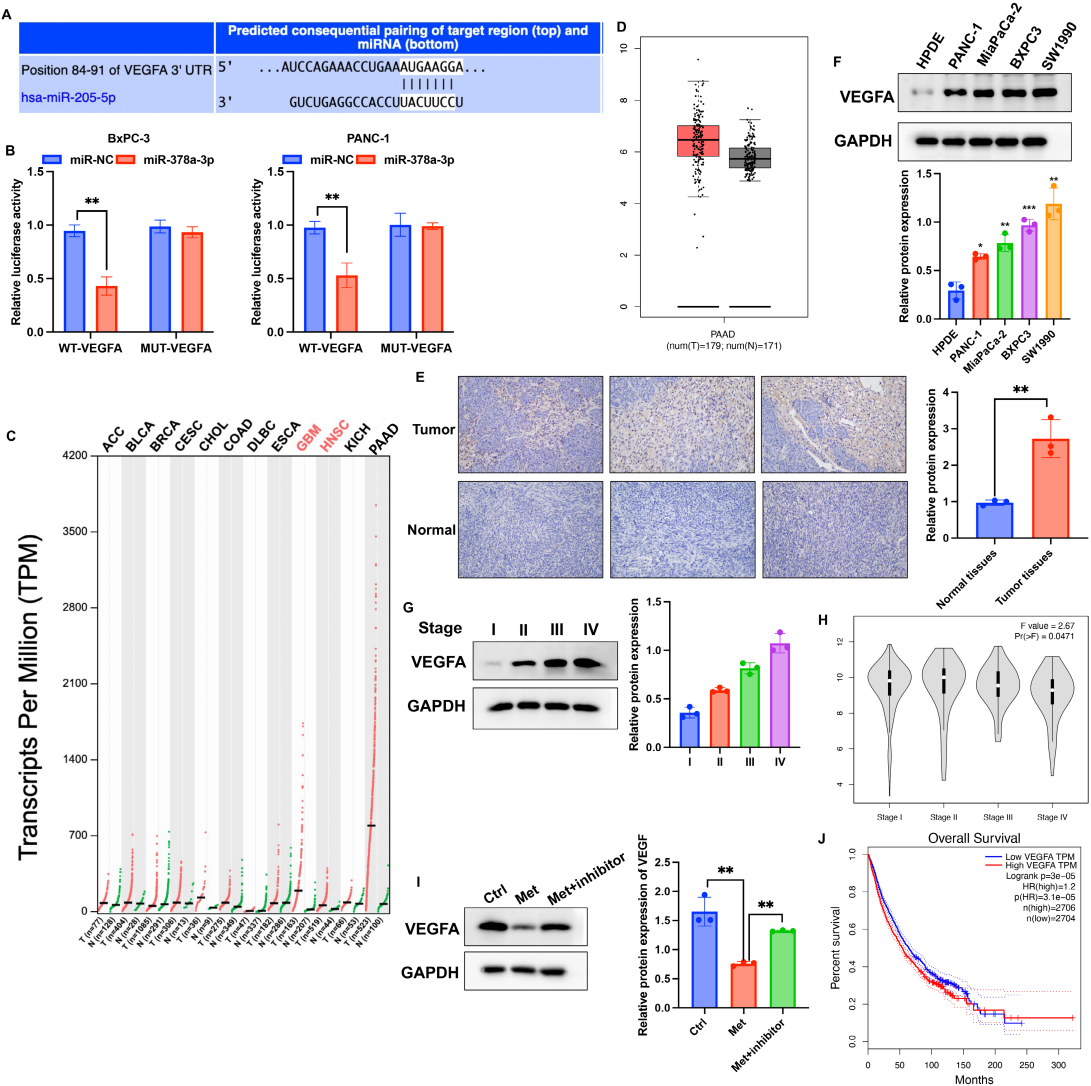
VEGFA is the potential regulator molecule of PC and the downstream target of miR‐378a‐3p. (A, B) Prediction and validation of the binding sites between miR‐378a‐3p and VEGFA. (C, D) Analysis of VEGFA expression in tumor and normal tissues through GEPIA and TCGA databases. (E, F) Detection of VEGFA expression in tumor tissues, normal tissues, PC cell lines, and normal pancreatic cells. (G, H) Examination of VEGFA expression in tumor tissues at different cancer stages. (I) Evaluation of the impact of the miR‐378a‐3p inhibitor on VEGFA expression. (J) Survival analysis based on VEGFA expression in PC patients from the TCGA database. Significance levels are denoted as (***, *p* < 0.001; **, *p* < 0.01; *, *p* < 0.05).

To further investigate the mechanism of action of miR‐378a‐3p in PC, we screened for its downstream target molecules. We identified specific binding regions between miR‐378a‐3p and VEGFA and confirmed the interaction (Figure 3A,B). We then explored whether VEGFA serves as a potential regulatory gene in PC. Based on GEPIA and TCGA databases, VEGFA expression was significantly upregulated in PC tissues (Figure 3C,D). Subsequently, we examined the expression in tumor tissues from PC patients and different PC cell lines. VEGFA exhibited significant overexpression in tumor tissues and PC cell lines (Figure 3E,F). Furthermore, the expression of VEGFA was positively correlated with tumor staging (Figure 3G,H), which are in line with the data from PC patients in our hospital (Table 1). Interestingly, Met significantly decreased the expression of VEGFA in BxPC3 cells, but after transfection with the miR‐378a‐3p inhibitor, VEGFA expression was significantly increased (Figure 3I). Elevated expression of VEGFA indicated a poorer prognosis (Figure 3J).

**TABLE 1:** Relationship between VEGF expression and characteristics in pancreatic cancer patients.

Features	Number of patients	High expression	Low expression	*p*
All cases	116	66	50	
Gender				0.216
Male	71	31	40	
Female	45	22	23	
Age				0.411
< 60	44	21	23	
≥ 60	72	32	40	
Smoking status				0.061
Non‐smoker	65	28	37	
Smoker	51	22	29	
T stage				0.015*
T1, T2	74	32	42	
T3, T4	42	22	20	
N stage				0.011*
N 0	76	35	41	
N1–N3	40	19	21	
M stage				0.032*
M 0	67	31	36	
M 1	49	23	26	

* < 0.05.

### 3.4 VEGFA Interacted With RGC‐32, and Involved in the Regulation of PC

**FIGURE 4:**
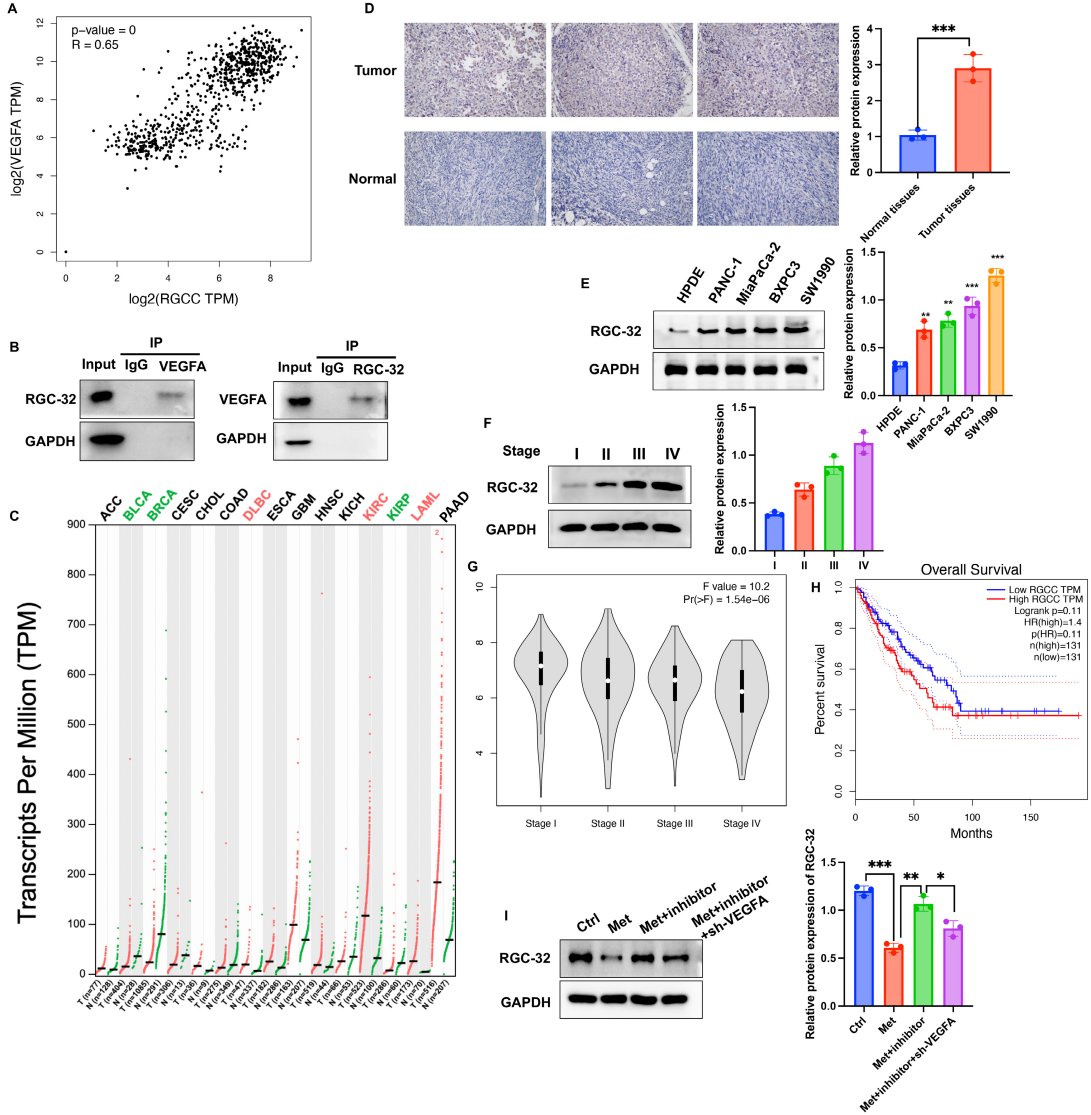
VEGFA interacted with RGC‐32, and involved in the regulation of PC. (A) Analyzed the correlation of VEGFA and RGC‐32 expression in PC tissues based on data from the GEPIA and TCGA databases. (B) Investigated the interaction between VEGFA and RGC‐32 through immunoprecipitation. (C) Analysis of RGC‐32 expression in tumor and normal tissues through GEPIA and TCGA databases. (D, E) Detection of RGC‐32 expression in tumor tissues, normal tissues, PC cell lines, and normal pancreatic cells. (F, G) Examination of RGC‐32 expression in tumor tissues at different cancer stages. (H) Survival analysis based on RGC‐32 expression in PC patients from the TCGA database. (I) Evaluation of the impact of the sh‐VEGFA on RGC‐32 expression. Significance levels are denoted as (***, *p* < 0.001; **, *p* < 0.01; *, *p* < 0.05).

Researches have reported that both VEGFA and RGC‐32 play crucial roles in angiogenesis. Furthermore, through analysis of the GEPIA database, we observed a significant positive correlation in the expression of VEGFA and RGC‐32 in PC tumor tissues (Figure 4A). Subsequently, we confirmed the interaction between VEGFA and RGC‐32 through immunoprecipitation methods (Figure 4B). The expression of RGC‐32 in tumor tissues and PC cell lines was markedly higher than that in adjacent tissues and normal pancreatic cells (Figure 4C–E). Moreover, the expression levels of RGC‐32 were positively correlated with the tumor staging of PC (Figure 4F,G). Elevated RGC‐32 expression indicated a poorer prognosis (Figure 4H). Furthermore, we found that Met significantly inhibited the expression of RGC‐32 in BxPC3 cells, but the miR‐378a‐3p inhibitor reversed the effects of Met, promoting RGC‐32 expression (Figure 4I). Additionally, transfection with sh‐VEGF further reduced the expression of RGC‐32, suggesting that RGC‐32 might be a downstream target of VEGF.

### 3.5 Knockdown of VEGFA Greatly Reversed the Influence of miR‐378a‐3p Inhibitor on Tumor Growth and Epithelial‐Mesenchymal Transition (EMT) Process

**FIGURE 5:**
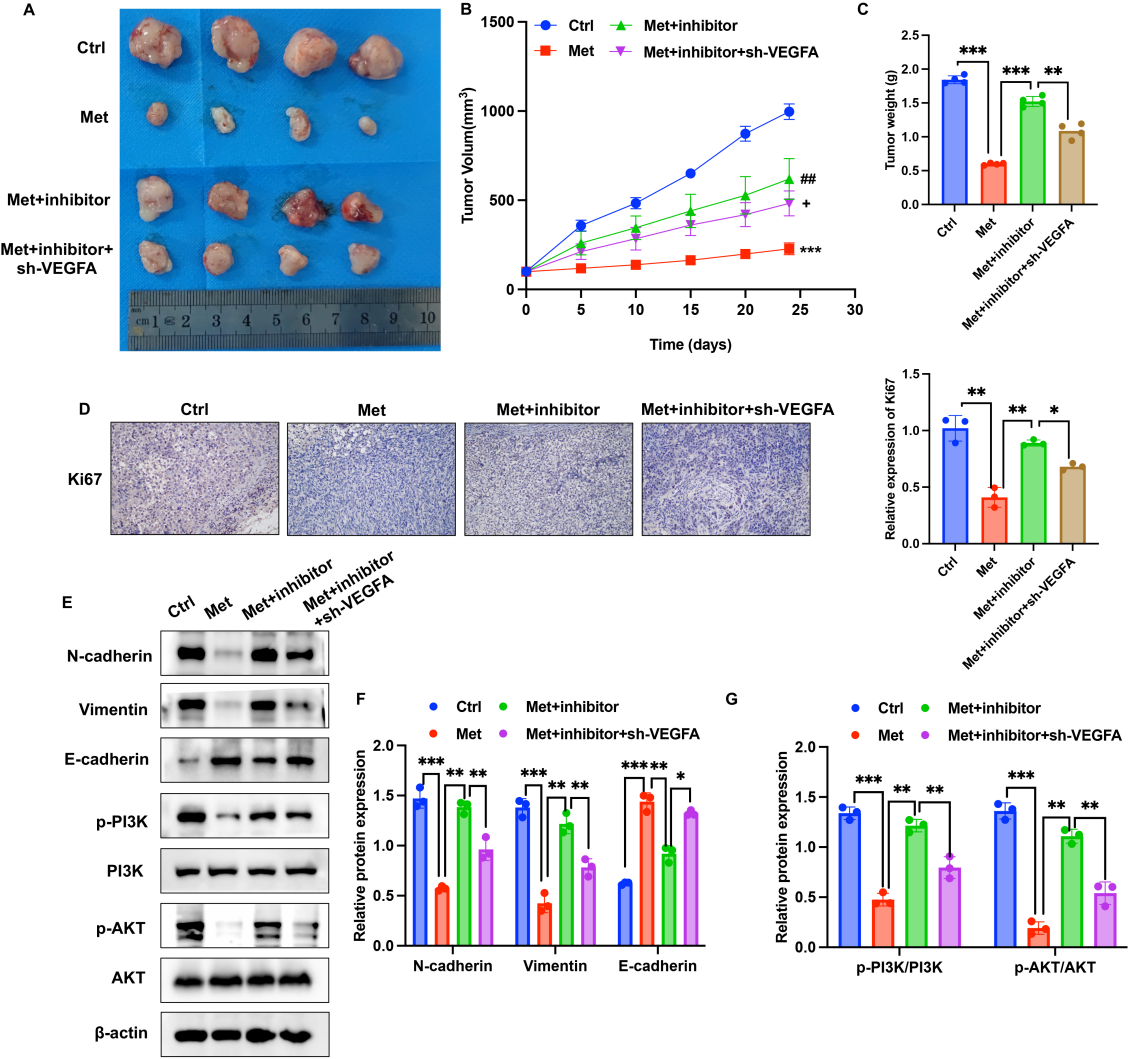
Knockdown of VEGFA greatly reversed the influence of miR‐378a‐3p inhibitor on tumor growth and EMT process in vivo. (A–C) Investigated the effects of Met, sh‐VEGFA, and miR‐378a‐3p inhibitor on tumor growth using a murine metastatic tumor model. (D) Detected the expression of Ki67 in tumor tissues through immunohistochemistry. (E–G) Examined the expression of EMT‐related proteins and the PI3K/AKT pathway using Western blotting. Significance levels are denoted as (***, *p* < 0.001; **, *p* < 0.01; *, *p* < 0.05).

To further investigate the regulatory roles of VEGFA and miR‐378a‐3p in PC, sh‐VEGFA and miR‐378a‐3p inhibitor were employed in the treatment of mice with metastatic tumors. The study revealed that Met significantly inhibited tumor growth, but the miR‐378a‐3p inhibitor reversed the tumor‐suppressive effect of Met. However, sh‐VEGFA further suppressed tumor growth (Figure 5A–C). This suggests that VEGFA might be a downstream effector of miR‐378a‐3p. Ki67 expression in tumor tissues was examined, and in the Met group, Ki67 expression significantly decreased. Conversely, after knocking down miR‐378a‐3p, Ki67 expression increased significantly, while sh‐VEGFA treatment led to its suppression (Figure 5D). The EMT process and the PI3K/AKT signaling pathway are widely implicated in tumor initiation, progression, and metastasis. We found that Met treatment suppressed the EMT process, accompanied by a decrease in the expression of N‐cadherin and Vimentin and an increase in E‐cadherin expression. Additionally, Met significantly inhibited the PI3K/AKT signaling pathway (Figure 5E–G). However, compared to the Met group, knocking down miR‐378a‐3p enhanced the EMT process and PI3K/AKT signaling pathway. Still, sh‐VEGFA reversed the effects of the miR‐378a‐3p inhibitor. This further confirms that Met might exert its inhibitory effect on PC through the regulation of miR‐378a‐3p/VEGFA.

### 3.6 Knockdown of VEGFA Greatly Reversed the Influence of miR‐378a‐3p Inhibitor on Cell Viability and EMT Process of BxPC3 Cells

**FIGURE 6:**
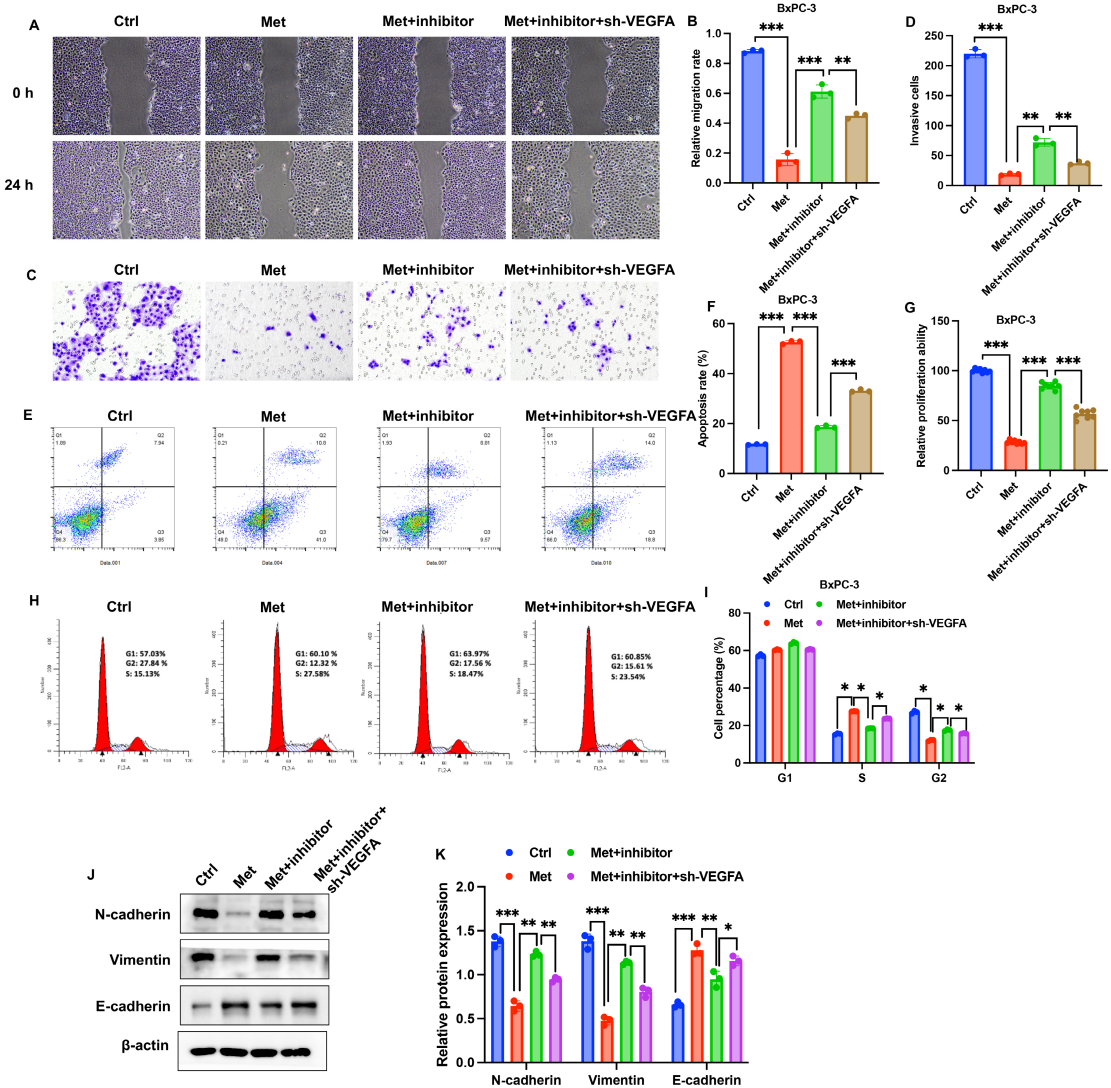
Knockdown of VEGFA greatly reversed the influence of miR‐378a‐3p inhibitor on cell viability and EMT process of BxPC3 cells. (A, B) Investigated cell migration through a wound healing assay. (C, D) Examined cell invasion using the Transwell method. (E–I) Evaluated cell apoptosis and cell cycle through flow cytometry, and detection of cell proliferation by CCK8 assay. (J) Examined the expression of EMT‐related proteins using Western blotting. Significance levels are denoted as (***, *p* < 0.001; **, *p* < 0.01; *, *p* < 0.05).

To further validate the regulatory role of VEGFA in PC, miR‐378a‐3p inhibitor and sh‐VEGFA were co‐transfected into BxPC3 cells. We observed that the miR‐378a‐3p inhibitor reversed the regulatory effects of Met on cell migration (Figure 6A,B), invasion (Figure 6C,D), apoptosis (Figure 6E,F), proliferation (Figure 6G), cell cycle (Figure 6H,I), and EMT process (Figure 6J,K). Furthermore, knocking down VEGFA inhibited cell migration, invasion, and proliferation, while inducing apoptosis compared to the Met+inhibitor group. Additionally, sh‐VEGFA significantly increased the proportion of cells in the S phase and suppressed the EMT process (Figure 6H–K).

## 4 Discussion

In recent years, the anti‐tumor effects of Met have become a focus of research. It has been reported that compared to other antidiabetic drugs, Met significantly reduces the risk of cancer and cancer‐related mortality in type 2 diabetes patients [15]. Furthermore, studies suggest that Met can improve the survival rates of patients with colorectal cancer, breast cancer, ovarian cancer, endometrial cancer, and others [16]. Several research findings indicate that Met significantly reduces the risk of PC in diabetic patients and extends the survival period of PC patients [17]. The mechanism of Met's anti‐PC effects is gradually being explored [18] but has not been fully clarified. In this study, we also found that Met significantly suppressed the proliferation, migration, and invasion of PANC‐1 and BxPC3 cells while promoting cell apoptosis (Figure 2B–H), which are in line with previous findings [15].

VEGFA, in particular, has been extensively studied and is found to be overexpressed in a majority of human cancers [19]. The close association between elevated VEGFA levels and increased tumor invasiveness, angiogenesis, metastasis, recurrence, and poor prognosis makes VEGFA a promising target for anti‐cancer therapies [20]. It was reported that VEGFA could accelerate tumor expansion by stimulating angiogenic milieu, and increasing microvascular density and permeability [21, 22]. In the research, we found that knockdown of VEGFA could reverse the influence of miR‐378a‐3p inhibitor on the cell viability of PC cells and tumor growth. In this research, sh‐VEGFA presented inhibition effects on cell migration, invasion, proliferation of BxPC‐3 cells, indicating that VEGFA is a carcinogenic factor.

RGC‐32 has emerged as a crucial player in tumorigenesis [23]. Recent studies have demonstrated its widespread expression in various organ tissues, including the pancreas [24]. RGC‐32 participates in the regulation of physiological processes such as cell cycle progression and cellular differentiation [25]. However, its role in different types of tumors varies, adding complexity to our understanding. Studies have shown differential expression levels and functions of RGC‐32 in distinct tumor tissues, suggesting its context‐dependent impact on cancer development [26]. The interaction between RGC‐32 and VEGFA was validated (Figure 4B), and the positive correlation of their expression in the tumor tissues were also validated (Figure 4A). In addition, the regulation of RGC‐32 by Met, miR‐378a‐3p inhibitor, and sh‐VEGFA was also observed (Figure 4I).

EMT is a phenomenon where epithelial cells undergo a transition from epithelial to mesenchymal cell characteristics under specific physiological and pathological conditions [27]. EMT has been recognized as a crucial pathological process that promotes tumor infiltration and metastasis. Previous studies have confirmed that EMT promotes invasion and distant metastasis in PC, and inhibiting EMT is considered a candidate direction for PC treatment [28]. Intervening in EMT to block the transition of early PC to metastatic/advanced PC is a critical aspect of improving the treatment outcomes for cancer patients [29]. We found that the EMT process and PI3K/AKT signaling pathway were greatly inhibited by Met treatment (Figure 5E–G). However, the regulation of EMT process and PI3K/AKT by Met were affected by miR‐378a‐3p inhibitor and sh‐VEGFA, suggesting that Met might modulate the PC through regulating miR‐378a‐3p/VEGFA/RGC‐32 axis. In addition, RGC‐32 was reported to promote the proliferation of PC and accelerate EMT [30]. In this research, we observed the positive correlation between VEGFA and RGC‐32 (Figure 4A), and that sh‐VEGFA inhibited EMT process (Figure 5E–G). These findings suggest that RGC‐32 might affect EMT through VEGFA, which need further validation.

The strength of this study is that we systematically validated the regulatory role of miR‐378a‐3p/VEGFA/RGC‐32 in PC using bioinformatics, animal experiments, and cell experiments. However, there are still some weaknesses in this study. The relevant research conclusions need to be validated at the clinical level. Meanwhile, the research on the mechanism in this paper is still relatively shallow and needs further exploration. How Met treatment increases miR‐378 levels remain unclear. Another report indicated that Met increased miR‐378a‐3p expression in C2C12 myoblasts, and inhibition of miR‐378a‐3p was shown to impair Met's effect in ATP production [31], which indirectly supports the conclusion of this study.

At present, the diagnosis and treatment methods for PC still need improvement. This study confirmed the regulatory role of miR‐378a‐3p/VEGFA/RGC‐32 axis in PC at both animal and cell levels. Targeting miR‐378a‐3p/VEGFA/RGC‐32 axis may provide a new target for the prevention and treatment of PC. In addition, miR‐378a‐3p/VEGFA/RGC‐32 also has the potential to become a biomarker for predicting PC occurrence.

## 5 Conclusion

We experimentally confirmed the inhibitory effects of Met on PC at both in vivo and in vitro levels. Using bioinformatics tools, we validated the role of miR‐378a‐3p/VEGFA/RGC‐32 in PC and confirmed the regulatory effects of miR‐378a‐3p inhibitor and sh‐VEGFA on PC. Our study suggests that Met may exert its inhibitory effects on PC through the miR‐378a‐3p/VEGFA/RGC‐32 pathway. Strategies targeting the miR‐378a‐3p/VEGFA/RGC‐32 axis represent a novel avenue for the prevention and treatment of PC.

## Author Contributions

**Jinli He:** conceptualization (equal), formal analysis (equal), project administration (lead), visualization (equal). **Yixing Luo:** data curation (equal), methodology (equal), project administration (equal). **Ying Ding:** methodology (equal), project administration (equal), supervision (equal), visualization (equal). **Liang Zhu:** conceptualization (equal), project administration (equal), writing – original draft (lead), writing – review and editing (lead).

## Ethics Statement

Ethical approval was obtained from the institutional ethics committee of the First Affiliated Hospital, Jiangxi Medical College, Nanchang University (approval no. CDYFY‐IACUC‐202302QR073).

## Consent

The authors have nothing to report.

## Conflicts of Interest

The authors declare no conflicts of interest.

## Permission to Reproduce Material From Other Sources

The authors have nothing to report.

## Data Availability

The data and material used to support the findings of this study are included within the manuscript and Supporting Information.

## References

[1] X. Lv , Z. Li , Y. Dai , et al., “The Mir‐199b‐5p Encapsulated in Adipocyte‐Derived Exosomes Mediates Radioresistance of Colorectal Cancer Cells by Targeting JAG1,” Heliyon 10, no. 2 (2024): e24412, 10.1016/j.heliyon.2024.e24412. 38293473 PMC10826727

[2] Y. Chen , J. Wang , Y. Huang , et al., “An Oncolytic System Produces Oxygen Selectively in Pancreatic Tumor Cells to Alleviate Hypoxia and Improve Immune Activation,” Pharmacological Research 199 (2024): 107053, 10.1016/j.phrs.2023.107053. 38176529

[3] Z. Zheng , Y. Bian , Y. Zhang , G. Ren , and G. Li , “Metformin Activates AMPK/SIRT1/NF‐kappaB Pathway and Induces Mitochondrial Dysfunction to Drive caspase3/GSDME‐Mediated Cancer Cell Pyroptosis,” Cell Cycle 19, no. 10 (2020): 1089–1104, 10.1080/15384101.2020.1743911. 32286137 PMC7217368

[4] B. Viollet , B. Guigas , N. Sanz Garcia , J. Leclerc , M. Foretz , and F. Andreelli , “Cellular and Molecular Mechanisms of Metformin: An Overview,” Clinical Science 122, no. 6 (2012): 253–270, 10.1042/CS20110386. 22117616 PMC3398862

[5] M. Ma , C. Ma , P. Li , et al., “Low Glucose Enhanced metformin's Inhibitory Effect on Pancreatic Cancer Cells by Suppressing Glycolysis and Inducing Energy Stress via Up‐Regulation of miR‐210‐5p,” Cell Cycle 19, no. 17 (2020): 2168–2181, 10.1080/15384101.2020.1796036. 32718270 PMC7513847

[6] E. T. Y. Mok , J. L. Chitty , and T. R. Cox , “miRNAs in Pancreatic Cancer Progression and Metastasis,” Clinical & Experimental Metastasis 41 (2024): 163–186, 10.1007/s10585-023-10256-0. 38240887 PMC11213741

[7] Y. S. Lee and A. Dutta , “MicroRNAs in Cancer,” Annual Review of Pathology 4 (2009): 199–227, 10.1146/annurev.pathol.4.110807.092222. PMC2769253 18817506

[8] M. V. Mencucci , M. C. Abba , and B. Maiztegui , “Decoding the Role of microRNA Dysregulation in the Interplay of Pancreatic Cancer and Type 2 Diabetes,” Molecular and Cellular Endocrinology 583 (2023): 112144, 10.1016/j.mce.2023.112144. 38161049

[9] Y. Dai , W. Shi , Y. Qiu , T. Xu , J. Lin , and Y. Su , “Circ_0000033 Up‐Regulates NUAK2 by Sequestering miR‐378a‐3p to Promote Breast Tumorigenesis,” Environmental and Molecular Mutagenesis 64, no. 6 (2023): 359–370, 10.1002/em.22558. 37357410

[10] L. Liu , S. Han , X. Xiao , et al., “Glucocorticoid‐Induced microRNA‐378 Signaling Mediates the Progression of Pancreatic Cancer by Enhancing Autophagy,” Cell Death & Disease 13, no. 12 (2022): 1052, 10.1038/s41419-022-05503-3. 36535942 PMC9763328

[11] P. Mabeta and V. Steenkamp , “The VEGF/VEGFR Axis Revisited: Implications for Cancer Therapy,” International Journal of Molecular Sciences 23, no. 24 (2022): 15585, 10.3390/ijms232415585. 36555234 PMC9779738

[12] S. I. Vlaicu , A. Tatomir , V. Rus , and H. Rus , “Role of C5b‐9 and RGC‐32 in Cancer,” Frontiers in Immunology 10 (2019): 1054, 10.3389/fimmu.2019.01054. 31156630 PMC6530392

[13] S. I. Vlaicu , A. Tatomir , F. Anselmo , et al., “RGC‐32 and Diseases: The First 20 Years,” Immunologic Research 67, no. 2–3 (2019): 267–279, 10.1007/s12026-019-09080-0. 31250246

[14] M. Brocard , S. Khasnis , C. D. Wood , C. Shannon‐Lowe , and M. J. West , “Pumilio Directs Deadenylation‐Associated Translational Repression of the Cyclin‐Dependent Kinase 1 Activator RGC‐32,” Nucleic Acids Research 46, no. 7 (2018): 3707–3725, 10.1093/nar/gky038. 29385536 PMC5909466

[15] H. Yamana , K. Kato , H. Kobara , et al., “Metformin Inhibits Proliferation and Tumor Growth of QGP‐1 Pancreatic Neuroendocrine Tumor Cells by Inducing Cell Cycle Arrest and Apoptosis,” Anticancer Research 40, no. 1 (2020): 121–132, 10.21873/anticanres.13933. 31892560

[16] Y. Li , L. Li , G. Zhang , et al., “Crucial microRNAs and Genes in Metformin's Anti‐Pancreatic Cancer Effect Explored by microRNA‐mRNA Integrated Analysis,” Investigational New Drugs 36, no. 1 (2018): 20–27, 10.1007/s10637-017-0508-2. 28875433

[17] K. Kato , H. Iwama , T. Yamashita , et al., “The Anti‐Diabetic Drug Metformin Inhibits Pancreatic Cancer Cell Proliferation In Vitro and In Vivo: Study of the microRNAs Associated With the Antitumor Effect of Metformin,” Oncology Reports 35, no. 3 (2016): 1582–1592, 10.3892/or.2015.4496. 26708419

[18] R. Tanaka , M. Tomosugi , M. Horinaka , Y. Sowa , and T. Sakai , “Metformin Causes G1‐Phase Arrest via Down‐Regulation of MiR‐221 and Enhances TRAIL Sensitivity Through DR5 Up‐Regulation in Pancreatic Cancer Cells,” PLoS One 10, no. 5 (2015): e0125779, 10.1371/journal.pone.0125779. 25955843 PMC4425682

[19] H. Al Kawas , I. Saaid , P. Jank , et al., “How VEGF‐A and Its Splice Variants Affect Breast Cancer Development—Clinical Implications,” Cellular Oncology 45, no. 2 (2022): 227–239, 10.1007/s13402-022-00665-w. 35303290 PMC9050780

[20] S. Ghalehbandi , J. Yuzugulen , M. Z. I. Pranjol , and M. H. Pourgholami , “The Role of VEGF in Cancer‐Induced Angiogenesis and Research Progress of Drugs Targeting VEGF,” European Journal of Pharmacology 949 (2023): 175586, 10.1016/j.ejphar.2023.175586. 36906141

[21] L. Claesson‐Welsh and M. Welsh , “VEGFA and Tumour Angiogenesis,” Journal of Internal Medicine 273, no. 2 (2013): 114–127, 10.1111/joim.12019. 23216836

[22] Y. Tian , X. Gao , X. Yang , S. Chen , and Y. Ren , “VEGFA Contributes to Tumor Property of Glioblastoma Cells by Promoting Differentiation of Myeloid‐Derived Suppressor Cells,” BMC Cancer 24, no. 1 (2024): 1040, 10.1186/s12885-024-12803-8. 39174921 PMC11342494

[23] P. Zhao , B. Wang , Z. Zhang , W. Zhang , and Y. Liu , “Response Gene to Complement 32 Expression in Macrophages Augments Paracrine Stimulation‐Mediated Colon Cancer Progression,” Cell Death & Disease 10, no. 10 (2019): 776, 10.1038/s41419-019-2006-2. 31601783 PMC6786990

[24] J. Zhang , J. R. Lei , L. L. Yuan , R. Wen , and J. Yang , “Response Gene to Complement‐32 Promotes Cell Survival via the NF‐kappaB Pathway in Non‐Small‐Cell Lung Cancer,” Experimental and Therapeutic Medicine 19, no. 1 (2020): 107–114, 10.3892/etm.2019.8177. 31853279 PMC6909658

[25] Z. H. Yang , J. Li , W. Z. Chen , and F. S. Kong , “Oncogenic Gene RGC‐32 Is a Direct Target of miR‐26b and Facilitates Tongue Squamous Cell Carcinoma Aggressiveness Through EMT and PI3K/AKT Signalling,” Cell Biochemistry and Function 38, no. 7 (2020): 943–954, 10.1002/cbf.3520. 32325539

[26] P. Zhao , D. Gao , Q. Wang , et al., “Response Gene to Complement 32 (RGC‐32) Expression on M2‐Polarized and Tumor‐Associated Macrophages Is M‐CSF‐Dependent and Enhanced by Tumor‐Derived IL‐4,” Cellular & Molecular Immunology 12, no. 6 (2015): 692–699, 10.1038/cmi.2014.108. 25418473 PMC4716617

[27] A. Dongre and R. A. Weinberg , “New Insights Into the Mechanisms of Epithelial‐Mesenchymal Transition and Implications for Cancer,” Nature Reviews. Molecular Cell Biology 20, no. 2 (2019): 69–84, 10.1038/s41580-018-0080-4. 30459476

[28] Y. Zhang and R. A. Weinberg , “Epithelial‐To‐Mesenchymal Transition in Cancer: Complexity and Opportunities,” Frontiers in Medicine 12, no. 4 (2018): 361–373, 10.1007/s11684-018-0656-6. PMC6186394 30043221

[29] P. Debnath , R. S. Huirem , P. Dutta , and S. Palchaudhuri , “Epithelial‐Mesenchymal Transition and Its Transcription Factors,” Bioscience Reports 42, no. 1 (2022): BSR20211754, 10.1042/BSR20211754. 34708244 PMC8703024

[30] L. Zhu and Y. Ding , “RGC‐32 Induces Transition of Pancreatic Cancer to Epithelial Mesenchyme In Vivo,” Pancreatology 18, no. 5 (2018): 572–576, 10.1016/j.pan.2018.05.480. 29886073

[31] I. F. Machado , J. S. Teodoro , A. C. Castela , C. M. Palmeira , and A. P. Rolo , “miR‐378a‐3p Participates in Metformin's Mechanism of Action on C2C12 Cells Under Hyperglycemia,” International Journal of Molecular Sciences 22, no. 2 (2021): 541, 10.3390/ijms22020541. 33430391 PMC7827403

